# Cancer stem cells in neuroblastoma therapy resistance

**DOI:** 10.20517/cdr.2019.72

**Published:** 2019-12-19

**Authors:** Natarajan Aravindan, Drishti Jain, Dinesh Babu Somasundaram, Terence S. Herman, Sheeja Aravindan

**Affiliations:** ^1^Department of Radiation Oncology, The University of Oklahoma Health Sciences Center, Oklahoma City, OK 73104, USA.; ^2^Department of Pathology, The University of Oklahoma Health Sciences Center, Oklahoma City, OK 73104, USA.; ^3^Department of Anesthesiology, The University of Oklahoma Health Sciences Center, Oklahoma City, OK 73104, USA.; ^4^Stephenson Cancer Center, Oklahoma City, OK 73104, USA.

**Keywords:** Neuroblastoma, therapy resistance, cancer stem cells, clonal selection, drug resistance, induced cancer stem cell

## Abstract

Neuroblastoma (NB) is the most common cancer of infancy and accounts for nearly one tenth of pediatric cancer deaths. This mortality rate has been attributed to the > 50% frequency of relapse despite intensive, multimodal clinical therapy in patients with progressive NB. Given the disease’s heterogeneity and developed resistance, attaining a cure after relapse of progressive NB is highly challenging. A rapid decrease in the timeline between successive recurrences is likely due to the ongoing acquisition of genetic rearrangements in undifferentiated NB-cancer stem cells (CSCs). In this review, we present the current understanding of NB-CSCs, their intrinsic role in tumorigenesis, their function in disease progression, and their influence on acquired therapy resistance and tumor evolution. In particular, this review focus on the intrinsic involvement of stem cells and signaling in the genesis of NB, the function of pre-existing CSCs in NB progression and therapy response, the formation and influence of induced CSCs (iCSCs) in drug resistance and tumor evolution, and the development of a CSC-targeted therapeutic approach.

## Introduction

Neuroblastoma (NB), an extracranial solid tumor, is the most common cancer at infancy (28%)^[1,2]^ and accounts for 6% of all pediatric cancers^[3-5]^. Clinically, NB disease progression is branded with hematogenous metastasis and frequent relapses, with a rapidly decreasing survival timeline (1st relapse in 18 months, 2nd relapse in 8.7 months, and 3rd relapse in 3.8 months)^[6,7]^. Given the disease’s heterogeneity and developed resistance, attaining a cure after relapse of progressive NB is highly challenging^[3,4,8,9]^. The rapid reduction in the timeline between successive recurrences^[6,7]^ is likely due to the ongoing acquisition of genetic rearrangements in undifferentiated cancer stem cells (CSCs)^[10-14]^ with intensive multi-modal clinical therapy (IMCT). The IMCT for high-risk NB comprises induction phase with alternating regimens of high-dose chemotherapeutic drugs [cyclophosphamide, cisplatin or carboplatin, vincristine, doxorubicin (adriamycin), etoposide, topotecan] and load reduction surgery; consolidation phase with more intensive chemotherapy (carboplatin, etoposide, topotecan, busulfan and melphalan, thiotepa) along with radiotherapy (external beam RT, MIBG RT) and stem cell transplant [autologous bone marrow transplantation (ABMT); peripheral blood stem cell reinfusion] and maintenance phase with retinoid drug treatment (13-cis-retinoic acid, isotretinoin), immunotherapy (dinutuximab), and immune-activating cytokine (GM-CSF, IL-2) treatment (www.cancer.org/cancer/neuroblastoma/treating.html; www.childrensoncologygroup.org/index.php/in-treatment-for-neuroblastoma). Appropriately, current studies are focused on understanding the acquired resistance after IMCT, particularly in determining the ongoing acquisitions of genetic/molecular rearrangements in therapy-resistant clones, CSC clonal selection/enrichment, and epithelial-to-mesenchymal transition (EMT) and phenotypic switch endorsing *de novo* evolution of CSCs^[15,16]^. Nevertheless, studies have affirmed that the presence of NB-CSCs not only indicates the progressive state of the disease, but also dictates poor response to therapy and poor clinical outcomes^[17,18]^. In this review, we aim to present the current understanding of NB-CSCs in disease progression, principally in the context of orchestrated resistance to IMCT.

## Relevance of stem cells in NB genesis

NB genesis, in general, is considered the accumulation of several mutations in actively dividing cells that form the nervous system during embryogenesis. The heterogeneity of NB indicates the presence of multipotent cells within the tumor, which could be the result of progenitor cell dedifferentiation under anomalous conditions or by accumulation of oncogenic mutations. Such an hypothesis took a definitive turn with the discovery of neural multipotent cells in the adult nervous system^[19]^, recognizing that mutations accumulated in stem cells or in defined progenitors as the trigger points for tumorigenesis. Embryogenesis, in which a single cell evolves into billions, is regarded as the cancer-prone period; indeed, many neural cancers, including NB, are more frequent in infants/children than in adults^[20,21]^. During embryogenesis, programmed cross-talk between the ectodermal bone morphogenetic protein 4 (BMP4) and notochord noggin and chordin leads to neural tube development and neural crest cell (NCC) migration, which later forms the peripheral nervous system (discussed in detail by de Weille^[22]^). The downstream fate (peripheral autonomic ganglia, neurons, glia cells, Schwann cells, adrenal medulla, melanocytes, thyroid parafollicular cells, and smooth muscle cells) of NCCs is governed by the orchestrated interplay of sonic hedgehog, BMP, snail family transcriptional repressor 2 (Slug), Snail, fibroblast growth factor (FGF), and wingless-related integration site (Wnt) signaling^[23]^.

**Figure 1:**
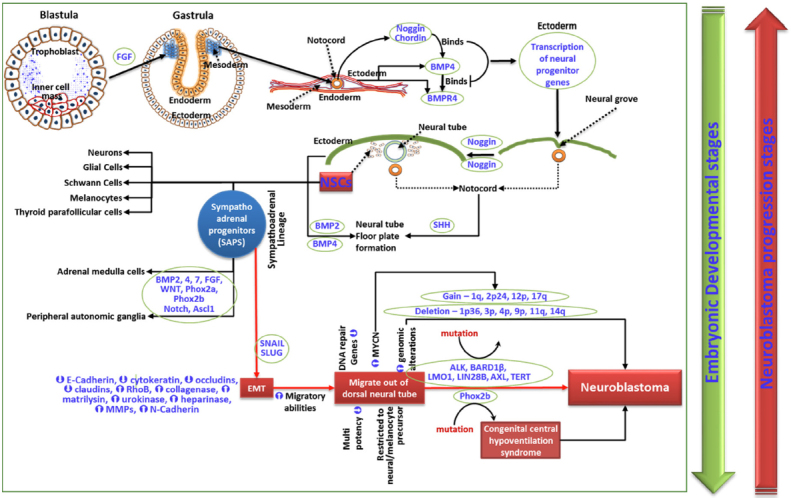
Schematic representation of cellular and molecular events in embryogenesis and diverted signaling events leading to NB genesis. SAPs undergo a Snail/Slug-dependent EMT that augments NCCs’ migratory abilities, allowing them to migrate out of the neural tube. The prompted migration, accompanied by regulation of DNA repair genes in SAPs, makes them vulnerable to many genomic alterations leading to the genesis of NB. While embryogenesis is a sequential step-down process from pluripotency to differentiation, advancing disease stages of NB progress successively from differentiated to undifferentiated self-renewing multipotent CSCs. NB: neuroblastoma; SAPs: sympathoadrenal progenitors; EMT: epithelial-mesenchymal transition; CSCs: cancer stem cells

A select subset of NCCs in the trunk region shows a sympathoadrenal lineage that contributes to the formation of sympathetic ganglia and medullary region of the adrenal gland. These committed NCCs are designated as sympathoadrenal progenitors (SAPs) and are believed to be the origin of NB [Figure 1]. SAPs constantly undergo a Snail/Slug-dependent EMT augmenting NCCs’ migratory abilities, allowing them to migrate out of the neural tube. The prompted migration is accompanied by DNA repair gene regulation in SAPs, making them vulnerable to genomic alterations^[24]^. SAPs lose part of their multipotency, and more are designated for neural or melanocyte lineage^[25]^. Bmp/Notch signals induce differentiation of these cells to the sympathetic ganglion chain. The SAPs express Phox2a/b, which is required for the production of enzymes in catecholamine biosynthesis. Downstream differentiation of SAPs is effected by a complex interplay of FGF, Notch, Wnt, achaete-scute BHLH-transcription factor (ASCL1), paired-like homeobox 2a (Phox2a), and Phox2b^[25]^. Phox2b mutations cause congenital central hypoventilation syndrome^[26]^, which serves as the predisposition for ganglioneuroma (GN), ganglioneuroblastoma (GNB), and NB. Since the morphologically more differentiated GN and GNB are lower grade with favorable outcomes, the genesis of aggressive NB depends on the stage of the sympathoadrenal progenitor (SAP), with un-/poorly-differentiated SAPs causing more lethal tumors. Accordingly, neuroblastic tumors could be Schwannian stroma-poor (undifferentiated, poorly differentiated, and differentiating), GNB intermixed Schwannian stroma-rich, or GN. Although the presence of catecholamines in an infant’s urine serves as the diagnostic marker for NB, its presence in high levels with much higher frequency than that of NB diagnosis (Dx)^[27,28]^ indicates a considerable spontaneous regression. In this regard, a mass screening study by Sawada assessing the levels of catecholamine metabolites, vanillylmandelic acid (VMA) and homovanillic acid (HVA), the sensitive diagnostic markers for NB in urine indicated that during the development of sympathetic neurons the incidence of in situ NB is higher than the incidence of sporadic cases^[28]^. Most of these in situ NBs spontaneously regress as the child ages. This is attributed to the VMA-to-HVA ratio where < 1 corresponds to a more aggressive, biologically primitive type of NBL associated with a shorter survival^[28]^. At the molecular level, NCCs that undergo EMT are characterized by the loss of epithelial morphology, regulation of junctional (E-cadherin, cytokeratin, occludins, claudins) complexes, and induction of migratory ras homolog family member B and matrix [collagenase, matrilysin, urokinase, heparanase, matrix metalloproteinases (MMPs), N-Cadherin] proteins. Consistently, aggressive cells from progressive NB exhibit similar physiognomies^[29]^. NB of progressive stages have been shown to generate self-renewing and multipotent CSCs that develop into neurons, Schwann-like cells, and melanocytes [Figure 1]^[17,30]^.

The magnificent biogenetic heterogeneity of NB indicates an interplay of more than one gene in tumor initiation and evolution. Researchers have identified that NB could be transmitted as recessive trait at low penetrance (e.g., locus 2p)^[31]^. Further studies validated the 2p23-24 locus with 104 genes, including v-myc avian myelocytomatosis viral oncogene NB derived homolog (*MYCN*)^[32]^. *MYCN*, the player in neurogenesis that is critical for expansion of progenitor cells, is overexpressed in NB^[33]^. Transgenic models of animal studies affirmed that overexpression of *MYCN* causes NB in their progeny^[29]^. In parallel, researchers also defined the association of the anaplastic lymphoma receptor tyrosine kinase (*ALK*) gene with the NB predisposition. Thus far, several germline and somatic *ALK* mutations have been identified (more frequent, *R1275, F1174, F1245*) in NB. These mutations promote receptor autophosphorylation in the kinase domain, leading to the activation of the ALK pathway. Likewise, genome wide association studies identified the association of *BRCA1* associated RING domain 1 β (BARD1β) with high-risk NB^[34]^. Other studies indicated that polymorphisms at locus of LIM domain only 1 (LMO1) [single nucleotide polymorphism (SNP) rs 2168101]^[35]^ and Lin-28 homolog B (LIN28B) are significantly involved in the susceptibility to NB^[36]^. Loss of chromosome 1p36 region, 3p, 4p, 9p, 11q, and 14q together with gain of 1q, 2p24, 12p, 17q, and mutations of *ALK* and AXL receptor tyrosine kinase (*AXL*) and telomerase reverse transcriptase (TERT) re-arrangements may orchestrate the transformation of normal NCCs to NB cells. Recent genome profiling studies in relapsed NB indicated new and acquired recurrent mutations in cadherin 5 (*CDH5*), dedicator of cytokinesis 8 (*DOCK8*), protein tyrosine phosphatase non-receptor 14 (*PTPN14*), HRas proto-oncogene (*HRAS*), and KRAS proto-oncogene (*KRAS*), signifying that these acquired mutations contribute to drug resistance and disease evolution^[37]^. However, it is not clear how and when the mutations occur, or whether these mutations occur in specific clones (e.g., CSCs) or enhance clonal selection and enrichment. In addition to the genetic causes, other factors, including manifestation of other neurocristopathies such as Hirshsprung’s disease^[38,39]^, Klippel-Feil syndrome, Waardenburg’s syndrome^[40]^, Ondine’s curse^[39]^, Beckwith-Weidemann syndrome^[41]^, Cushing’s syndrome^[42,43]^, fetal alcohol syndrome^[44-46]^, fetal hydantoin syndrome caused due to intake of anti-seizure drug phenylhydantoin^[47,48]^, and exposure to products causing high blood pressure, sweating, headache, and abnormal heartbeats in mothers, have been associated with increased susceptibility to NB.

## NB-CSCs in therapy resistance and disease evolution

Tumor heterogeneity, in general, has been explained by two models: the stochastic model, in which each cancer cell has the ability to contribute to the tumor evolution, and a more accepted CSC model, in which a small subset of cells within the tumor has high tumorigenic potential and can give rise to all other cells of the tumor^[49]^. Over the last two decades, the functional significance of CSCs in tumor initiation, progression, response to therapy, and poor clinical outcomes has been recognized in different types of tumors, including pancreatic cancer^[50]^, liver cancer^[51,52]^, lung cancer^[53]^, breast cancer^[54]^, head and neck squamous cell carcinoma^[55]^, colon cancer^[56,57]^, brain tumors^[58]^, leukemia^[59]^, and NB^[18,60,61]^. By definition, CSCs are the indefinitely proliferating subpopulation within the tumor, exhibiting stem-like properties that include self-renewal, multipotency and/or pluripotency maintenance, tumorigenic potential, an unparalleled metastatic state, and anti-apoptotic ability. CSCs are known to play roles in induced resistance to chemotherapy and radiation, resulting in an increased risk of tumor progression, relapse, and recurrence^[62-64]^. This function is attributed to the possession of multiple mechanisms, including repression of apoptosis, increased DNA damage repair, conserved dormancy, and altered drug response^[65]^. Traditional treatment modalities, i.e., conventional chemotherapy and radiotherapy, are adopted based on the stochastic model, with a focus on killing all cancer cells^[49]^. However, later studies indicated that the limitations of such a strategy are mainly attributed to the intrinsic resistance of the CSCs within the tumor^[16,66,67]^. The current understanding of the role of CSCs in therapy response, tumor evolution, and the development of CSC-targeted therapeutic strategies for various tumors was extensively reviewed [Table 1] and provided a strong basis for similar biology in NB.

**Table 1:** Documented reviews on the role of CSCs in therapy response, tumor evolution, and the development of CSC-targeted therapeutic strategies for diverse human tumors, including NB. These compilations clearly define the identification of CSC surface markers, orchestrated signaling events, influence of ECM and TME; portray the functions of CSCs in induced/acquired therapy resistance; and recognize the possibility and benefit of CSC-targeted therapies in cancer treatment

Title	Ref.
Chemoresistance, cancer stem cells, and miRNA influences: The case for neuroblastoma	Buhagiar and Ayers^[72]^
p53, stem cell biology and childhood blastomas	Oh *et al.*^[73]^
Cancer stem cells and pediatric solid tumors	Friedman and Gillespie^[74]^
Cancer stem cells and their interaction with the tumor microenvironment in neuroblastoma	Garner and Beierle^[75]^
Multidrug resistance and cancer stem cells in neuroblastoma and hepatoblastoma	Alisi *et al.*^[16]^
CD133: A stem cell biomarker and beyond	Li^[76]^
Cancer stem cells (CSCs) in drug resistance and their therapeutic implications in cancer treatment	Phi *et al.*^[77]^
Cancer stem cells revisited	Batlle and Clevers^[78]^
Mechanisms of chemoresistance in cancer stem cells	Abdullah and Chow^[15]^
Cancer stem cell metabolism	Pages *et al.*^[62]^
Cancer stem cells: Implications for cancer therapy	Dawood *et al.*^[79]^
Cancer stem cell surface markers on normal stem cells	Kim and Ryu^[80]^
Cancer stem cell signaling pathways	Matsui^[81]^
Cancer stem cells: Basic concepts and therapeutic implications	Nassar and Blanpain^[82]^
Cancer stem cells and tumorigenesis	Zhu and Fan^[64]^
The therapeutic promise of the cancer stem cell concept	Frank *et al.*^[83]^
Drug resistance driven by cancer stem cells and their niche	Vila *et al.*^[63]^
How to hit mesenchymal stromal cells and make the tumor microenvironment immunostimulant rather than immunosuppressive	Poggi *et al.*^[84]^
Tumor-derived spheroids: Relevance to cancer stem cells and clinical applications	Ishiguro *et al.*^[85]^
Therapeutic strategies targeting cancer stem cells	Yoshida and Saya^[86]^
Therapies targeting cancer stem cells: Current trends and future challenges	Dragu *et al.*^[49]^
Stem cell theory of carcinogenesis	Trosko and Chang^[87]^

In the context of progressive NB, several studies have identified that CSCs play a crucial and definite role in disease progression, relapse, and poor prognosis^[61]^. High-risk NBs consist of small populations of cells with preserved stemness characteristics. These clones exhibit the ability to form highly resistant tumorospheres and have high metastatic potential^[60]^. NB-CSCs are identified based on morphology, cell efflux property, cell surface markers, and tumorosphere formation in *in vitro* conditions. On a morphological basis, NB cells can be classified as neuroblastic [neuroblastic type cells (N-type), immature, tumorigenic], Schwann-like (S-type, non-tumorigenic with limited lifespan), and intermediate (I) type cells^[30,68]^. The malignant NCC-like intermediate type NB cells (I-type) cells are multipotent and can self-renew, as well as differentiate into N and S type morphologies^[69]^. Beyond the self-renewal and differentiation capabilities, I-type cells are associated with tumor relapse/metastasis^[69]^, possess high tumor-forming capacity *in vivo*^[70]^, and are characterized by overexpression of stemness-related molecules, including cluster of differentiation 133 (CD133), cKIT, NOTCH 1, GPCR class C group 5 member C (GPRC5C), and tropomyosin receptor kinase B (TRKB)^[71]^. In addition, the role of NOTCH 1 in maintaining the stem cell phenotype has been recognized. However, I-type cells are only regarded as aggressive NB cells with relatively high plasticity, and hence do not fully meet the criteria for categorization as CSCs.

### Drug efflux pumps and chemoresistance

Identification and characterization of NB-CSCs that could be useful for precisely assessing NB Dx/prognosis, therapy response, and clinical outcomes have been continuously evolving over the last two decades. Nestin and ABC subfamily G member 2 (ABCG2) are 2 markers that were regarded as NB-CSC markers very early on. The NB cells presented with drug efflux pumps (that exclude Hoescht dye) that confer multi-drug resistance (MDR) have been consistently identified as a “side-population” (SP). These cells were found to have high expression of ABCG2 and ABC subfamily A member 3 (ABCA3), and are highly [Table 1]. Documented reviews on the role of CSCs in therapy response, tumor evolution, and the development of CSC-targeted therapeutic strategies for diverse human tumors, including NB. These compilations clearly define the identification of CSC surface markers, orchestrated signaling events, influence of extracellular matrix (ECM) and tumor microenvironment (TME); portray the functions of CSCs in induced/acquired therapy resistance; and recognize the possibility and benefit of CSC-targeted therapies in cancer treatment [Table 1].

Concentrated in post-IMCT relapsed tumors (*vs.* pretreatment controls)^[88]^. ATP binding cassette (ABC) transporters are the transmembrane proteins that drive efflux of many chemotherapeutic drugs and hence dictate chemoresistance^[89,90]^. Newton *et al.*^[88]^ compared the Side populations derived from the same patients at diagnosis and at relapse after IMCT. Their findings showed significantly increased Side populations in post-IMCT relapsed NB (*vs.* at diagnosis), heightened proliferation and clonal expansion of drug-resistant Side populations, and acquired gain of pluripotency maintaining factors [nanog homeobox (Nanog), POU class 5 homeobox 1 (Oct3/4)] in drug-resistant Side populations. Such critical information clearly indicated the acquired stemness-related genetic alterations in Side populations, their selection and clonal expansion, that subsequently played a crucial role in chemoresistance and tumor relapse^[88]^. ABCG2, an ABC protein associated with neural stem and progenitor cells, has been shown to influence stemness maintenance. Nestin, on the other hand, is a neuronal stem cell protein that also serves as a putative marker for CSC^[91]^. In neural stem/progenitor cells, ABCG2 and Nestin exhibits heightened colocalization and in particular the ABCG2^**+**^ and Nestin^**+**^ cells mimic each other in their ability to form tumorospheres^[90]^. Consistently, number of studies designated them as putative CSC markers for various tumors including NB^[61,91,92]^. Similarly, the NB Side population cells expressing high levels of ABCG2 and ABCA3 transporter genes have been shown to possess high capabilities to expel chemotherapy drugs (e.g., mitoxantrone) and promote proliferation^[16]^. ABCG2 have also been shown to transport a number of common chemotherapy drugs, including anthracyclins, imatinib, and Topoisomerase I and II inhibitors^[16]^.

### Cell surface CSC markers and therapy resistance

Identifying the surface expression of select CSC markers clearly provides the basis of the CSC composition in NB as such and for drug response. Recognizing specific surface markers is useful for characterization of CSCs, examining NB biology/evolution, and therapeutic targeting. To date, a number of markers, including CD133, frizzled class receptor 6 (FZD6), leucine rich repeat containing GPCR 5 (LGR5), aldehyde dehydrogenase (ALDH), ALDH1A2, ALDH1A3, cluster of differentiation 114 (CD114), and cluster of differentiation 117 (C-kit), have been used in NB^[93]^.

#### CD133

NB-CSCs have been shown to pose a genetic profile different from that of non-stem tumor cells, including gains of 16p13.3, 19p13.3, and 19q13.33. Interestingly, the gain in 16p13 is significantly associated with the expression of CD133 in NB^[94]^. CD133 (Prominin-1), a pentaspan transmembrane protein expressed in neural stem cells, has been indicated as a marker for tumor-initiating cells^[76]^. CD133 levels were inversely correlated with overall survival (OS) of NB patients^[95]^. It has been shown that the poor association occurs through Serine/Threonine kinase (AKT) pathway-mediated chemoresistance^[96]^. Muting CD133 decreases NB cell colony formation and proliferation, increases differentiation *in vitro*, and decreases tumor burden *in vivo*^[97]^. On a therapy response note, studies have shown that CD133^**+**^ NB cells efficiently develop tumorospheres, which exhibited high resistance to doxorubicin (DOX) treatment with upmodulation of ABCG2^[61]^. Conversely, DOX treatment resulted in increased CD133- and ABCG2-expressing Side populations and the ability of these Side populations to generate non-Side population cells^[61]^ exhibiting: (1) drug resistance in CD133-expressing Side populations; (2) clonal selection and enrichment of drug-resistant CSCs; and (3) drug resistance and disease evolution mediated by CSCs. Consistently, studies have indicated the criticality of CD133-expressing Side populations in NB progression and therapy response. For instance, studies demonstrated that (1) non-adherent clumps of tumor cells that express CD133, OCT4, pAKT could grow in a serum-free medium with higher colony and neurosphere formation^[98]^; (2) CD133 levels were directly associated with NB advanced disease stages and inversely correlated with postoperative survival time^[95]^; and (3) CD133^**+**^ NB cells were more resistant to cisplatin, carboplatin, DOX, and etoposide (*vs.* CD133^**-**^ cells) and presented with increased phosphorylation of extracellular signal-regulated kinases (ERK) and P38 mitogen-activated protein kinases (P38)^[99]^. Consistently, an independent study showed that targeted inhibition of CD133 in NB cells produced increased RET expression and NB differentiation and, this response is mediated through the regulation of the p38 MAPK and phosphoinositide-3-kinase (PI3K)/AKT pathways^[97]^.

CD133^**+**^ NB-CSCs exhibit inhibited levels of mitogen-activated protein kinase phosphatase 1 (MKP1) and increased phosphorylation of ERK and P38^[99]^. The influence of these MAPKs in cell cycle progression, differentiation, and cell death has been extensively documented. It has been realized that chemotherapy drugs such as cisplatin activate JNK, ERK, and other MAPKs through multitude mechanisms that dictate the development of drug resistance^[100]^. Since MKP1 regulation leads to the phosphorylation of these kinases, the low levels of MKP1 in CD133^**+**^ cells directly relate to the induced drug resistance in NB^[99]^. However, CD133-targeted therapeutic measures for NB warrant in-depth investigation. For instance, although valproic acid (VPA), a histone deacetylase (HDAC) inhibitor, has been shown to induce cell death in NB cells and overcome hypoxia-induced resistance to cisplatin^[101]^, it has also been reported that VPA treatment increases the percentage of CD133^**+**^ NB-CSCs and does not induce apoptosis in these cells. Similarly, decitabine, a hypomethylating drug, and trichostatin A, a HDAC inhibitor, have been shown to induce surface expression of CD133 in CD133^**-**^ cancer cells^[66]^. Detailed insights on the regulation of CD133 expression and its role in self renewal, tumorigenesis, metastasis, chemo/radio-resistance, metabolism, dedifferentiation, and autophagy have been well documented^[76]^. Although CD133 is highly regarded as a CSC marker in NB settings, it is pertinent to note that CD133 is only expressed in ~40% of primary tumors, which precludes its use as a stand-alone NB-CSC marker.

#### FZD6, LGR5 and ALDH

Surface marker FZD6, a WNT receptor, is inversely associated with OS in patients with NB^[102]^. It has been shown that FZD6^**+**^ Side populations were selectively enriched in hypoxic regions, and such Side populations can readily form tumorospheres and develop more aggressive tumors. Likewise, LGR5, a WNT-responsive G-protein-coupled receptors (GPCR) protein, significantly correlates with poor event-free survival (EFS) in high-risk NB subsets^[103]^. LGR5 is specifically expressed in CSCs, is known to brace WNT/β-catenin signaling as an R-spondins receptor, and drives oncogenesis^[104]^. Elevated levels of LGR5 in IMCT-resistant cells were associated with aggressive phenotype, and cells presented with high LGR5 levels were highly chemoresistant^[104]^. Activation of LGR5 with WNT3a ligands promotes WNT-pathway-dependent proliferation. However, muting LRG5 has been shown to mediate apoptosis through mitogen-activated protein kinase (MEK)/ERK signaling, independent of the WNT pathway. Moreover, upmodulation of ALDHs was associated with retinoic acid (RA) tolerance^[105]^. Consistently, ALDH1A2 and ALDH1A3 showed an inverse relationship with OS in high-risk NB patients. Independent studies have demonstrated that silencing ALDH1A2 or ALDH1A3 reduced clonal expansion of tumor-initiating cells and tumorosphere formation^[106,107]^. Hartomo *et al.*^[106]^ assessed the activity and expression of 19 isoforms of ALDH, and identified that ALDH1A2, ALDH1L1, and ALDH3B2 expression consistently induced tumorosphere and colony formation. However, they also recognized that ALDH1A2 is the only candidate to show a significant association with poor prognosis of patients with NB. More importantly, high levels of ALDH1A2 expression correlated with the growth and dedifferentiation of NB xenografts, as well as RA treatment resistance, in NB cells^[106]^. It is critical to note that there is thus far no definitive *in vivo* evidence documented for FZD6-, LGR5-, or ALDH1A3-positive Side populations in terms of tumor evolution or drug-response in NB. However, the role of these candidates as drivers, players, or enhancers in acquired disease resistance and disease progression is possible and warrants further investigation. A study by Kuo *et al*.^[108]^ identified the role of jumonji, AT-rich interactive domain 1 (JARID1B) (KDM5B) a histone lysine demethylase, in modulating stemness-related signaling. High levels of JARID1B were associated with high levels of ALDH activity, and this upmodulation was associated with enriched NB tumorospheres. JARID1B-enriched SP showed the strongest sphere-forming capacity and also presented with high levels of Nestin colocalization. While JARID1B-expressing NB-CSCs have been shown to be less responsive to DOX, etoposide, and cisplatin, silencing JARID1B resulted in decreased invasiveness, CSC phenotype, EMT process, and compromised NOTCH signaling^[108]^.

#### CD114

Recent studies, identified the surface expression of CD114, a G-CSF receptor, as a putative marker for NB-CSCs. Unlike other surface markers, the CD114-expressing SP is < 1% and exhibit several CSC characteristics with 10 times more tumorigenic capacity^[109]^. More importantly, incidence of CD114^**+**^ Side populations has been reported in all NB cell lines, patient samples, and PDXs tested to date, with a frequency ranging from 0.01%-3%. A drift toward clonal selection and enrichment of CD114^**+**^ SP after IMCT in relapsed tumor and in metastases demonstrates the intrinsic chemoresistant capabilities of this SP^[109,110]^. Growing evidence has documented the characteristics of the CD114^**+**^ SP, including the near-identical gene profile [e.g., Sox10, twist family BHLH transcription (TWIST), vimentin (VIM), MMPs] to that of pre-/early migratory NCCs that maintain multipotent capacity^[111]^. Furthermore, studies have shown that NB pathogenesis and dissemination are dependent on CD114^**+**^ SP through downstream activation of signal transducer and activator of transcription 3 (*STAT3*) target genes^[110]^. In normal neural tissue, CD114 (G-CSFR) promotes neurogenesis and survival and expansion of the neural stem cells^[112,113]^. G-CSF activates STAT3 in these receptor-positive CSCs; this signaling promotes stemness maintenance, clonal expansion, tumor formation and dissemination, and chemoresistance^[109]^. The existence of a granulocyte colony-stimulating factor (GCSF)→STAT3→GCSF positive feedback cycle has also been recognized. Targeted deregulation of this feedback loop with STAT3 inhibitors not only depletes the CSC-SP within tumors, but also prompts tumor regression and profound chemosensitization^[110]^.

#### Other CSC markers involved in resistance

CFC1, a member of the epidermal growth factor-Cripto/FRL-1/Cryptic (EGF-CFC) family, with designated functions in embryonic development, has been recently recognized as a CSC marker for NB^[114]^. CFC1, is strongly expressed in sphere-forming CSCs and is associated with unfavorable prognosis in NB patients. In addition, the functional role of CFC1 in NB tumorosphere formation has been realized and further identified that CFC1 directly targets activin-A induced cell differentiation and Smad phosphorylation, resulting in tumor progression. Analysis of activin-A signaling further identified the inhibition of differentiation-inducing [BMP-4, transforming growth factor beta (TGFβ)-1, and TGFβ-3] and tumor suppressor [cyclin dependent kinase 4 (CDK4), cyclin dependent kinase inhibitor 2A (CDKN2A), p14-ARF, and p16-INK4A] molecules in CFC1-overexpressing cells^[114]^. Naiditch *et al.*^[67]^ assessed the differential regulation of genes in DOX-resistant NB cells compared with their wild-type counterparts. The high-throughput whole genome approach identified chemotherapy resistance-associated differential regulation of > 1500 candidates. With the network analysis, this study clearly identified the deregulation of EMT pathway, stemness maintenance, and tumor progression signaling. The regulation/deregulation of neuronal, epithelial, mesenchymal, and pluripotency maintenance markers in these DOX-resistant, highly invasive cells illustrates the role of acquired mesenchymal change in induced drug resistance.

B cell-specific Moloney murine leukemia virus integration site 1 (BMI1), a polycomb protein has been shown to have critical roles in stem cell self-renewal, initiation of cancer and chemoresistance in many human malignancies including NB^[115-117]^. BMI1 has been shown to be highly expressed in NB^[117]^ and is essential for the pathogenesis of the disease^[115]^. The binding of E2F-1 and MYCN^[118]^ to BMI1 promoter and its activation were documented^[117]^. It has been shown that BMI1 inflicts NB progression and contributes to therapy resistance/disease evolution through modulating key players in tumorigenesis including Cyclin E1, KIF1Bβ, TSLC1 and others^[119,120]^. More importantly, the role of BMI1 in the regulation self-renewal capacity and differentiation of I-type NB CSCs has been realized^[116]^. Consistently, Melone *et al*.^[121]^ indicated the clinical relevance of BMI1 in NB and defined its association to advanced disease stages. Though the direct role of BMI1^**+**^ NB-CSCs in orchestrating therapy resistance is currently unknown, targeting BMI1^**+**^ CSCs has been shown to overcome chemoresistance^[122-125]^ and radio-resistance^[126-128]^ in many human cancers.

### Induced CSCs and therapy resistance

As discussed above, drug resistance and cancer recurrence are majorly affected by the preexisting CSCs (tumor-initiating cells) that are derived from normal stem cells under certain environments. The overall hypothesis is that these preexisting CSCs cause therapy resistance and/or disease relapse with their unique abilities of clonal selection, self-renewal, clonal expansion, stemness maintenance, and plasticity. In parallel, recent findings indicated that CSCs can be formed from non-stem cancer cells exposed to radiation or chemotherapeutic drugs, creating an SP of induced-CSCs (iCSCs)^[129]^. The transformation of non-stem cancer cells into iCSCs involves reprogramming factors [OCT4, SOX2, myelocytomatosis viral oncogene homolog (C-MYC), kruppel like factor 4 (KLF4)]^[130-133]^ depended dedifferentiation. Such transformation always displays significant CSC properties, sphere formation, drug resistance, and tumorigenicity^[134,135]^. In addition to radiation and chemotherapy drugs, many other driving factors, including temperature, external cytokines/transformation factors, inhibitor of DNA binding 4 (ID4), and interleukins 6 (IL6) have been causally linked to dedifferentiation of non-stem cancer cells^[136-138]^. In NB, epigenetic modifiers have been shown to endorse iCSCs formation and maintenance^[139]^. With regard to the role of CSCs in induced drug resistance, studies across human tumors, including NB, widely agreed that CSCs are inherently resistant to radiotherapy and/or chemotherapy^[140-143]^. However, the understanding of the acquired genetic alterations in non-stem cancer cells and their transformation into iCSCs after radiochemotherapy is recently on an upsurge (reviewed in detail elsewhere^[144]^). ICSCs formation was documented with common chemotherapy drugs [fluorouracil (5-FU), DOX] and with various qualities of radiation. Assessment of the mechanism(s) involved in the formation of iCSCs revealed that a complex interplay of multiple signaling pathways, small non-coding RNAs (microRNAs), and the appropriate TME facilitates dedifferentiation. Interestingly, both the transformation of normal stem cells in to CSCs and dedifferentiation of non-stem cancer cells into iCSCs employ near-identical pathways (e.g., NFkB, Notch, Wnt, Hedgehog). During the formation of iCSCs from non-stem cancer cells exposed to radiotherapy, these signaling events have been shown to coincide with other complementary pathways, including pluripotency maintenance reprogramming (SOX-2, OCT4, NANOG) and plasticity, and to configure iCSCs.

Expressional deregulation of many other proteins are also causally linked to the dedifferentiation of non-stem cancer cells and formation of iCSCs^[118,145-147]^. For instance, Lamin A/C, the type V intermediate filaments of nuclear lamina, is often reduced or absent in proliferative cells of various tumors^[148,149]^. Lack of lamin A/C predisposes cells towards an immature phenotype and influences the presence of tumor-initiating cells in NB^[150]^. Selective silencing of lamin A/C triggers the formation of NB-iCSCs with self-renewing ability. Loss of lamin A/C is also MYCN expression-dependent^[150]^. SOX2, a member of the SRY-related high mobility group box, is a transcription factor that is mostly expressed during embryonic development. It has been shown to affect cell fate and differentiation^[151]^, self-renewal, and proliferation^[152-156]^. In NB, SOX2 levels were heightened and its expression correlated with advanced disease stage^[157]^. Studies with SOX2 stably over-expressed NB cells not only recognized amplified tumorigenicity, but also showed clonal selection and expansion of stem-like cells (with loss of N and S type). Selective silencing of SOX2 in highly malignant I-type CSCs greatly reduced its tumorigenicity and enriched N and S type cells^[158]^. Targeting SOX2 resulted in cell-cycle arrest at G0/G1 phase and hence drove decreased cell proliferation. Similarly, tailless-like receptors (TLX), which is a nuclear receptor and a transcription factor, plays a critical role in self-renewing, undifferentiated, and proliferative states of neural stem cells^[159]^. During NB genesis, PHOX2B dictates dedifferentiation of SAPs by upmodulating T cell leukemia homeobox 3 (TLX3) and neurotrophin receptor P75 (P75). TLX has been causally linked to NB dedifferentiation, resulting in tumorigenesis by activating MMP2 and MMP9. Consistently, TLX has been identified to be overexpressed in NB, involved in progression of tumorigenesis and correlated with shorter survival rates^[160]^.

### Role of TME in influencing CSC status and therapy resistance

The TME also serves as a critical regulator of stem cell differentiation, dedifferentiation of non-stem cancer cells, and tumorigenesis^[161,162]^. The TME contributes hypoxia, inflammation, acidic stress (pH), and remodeling of ECM, coordinates CSC self-renewal, and inhibits differentiation. For instance, NB exposure to hypoxia led to the upmodulation of NCC markers (C-Kit, Notch1) and hypoxia-induced response genes [hypoxia inducible factor *(HIF)-1α*, *HIF-2α*]^[163,164]^. Similarly, studies have showed a greater concentration of tumor associated macrophages (TAMs) in metastatic NB than in locoregional disease^[165]^. The role and functions of these microenvironmental factors are reviewed in detail elsewhere^[75]^. With regard to their function in orchestrated drug resistance and NB evolution, mesenchymal stromal cell-derived cancer-associated fibroblasts (CAF) contributed significantly to clonal expansion and chemoresistance. Once induced, CAFs maintain their activated state; increased CAFs were linked with induced microvascular proliferation and Schwannian stroma-poor histology. This study showed that the pro-tumorigenic activity of mesenchymal stem cells (MSC)-CAF occurs through the co-activation of the janus kinase 2 (JAK2)/STAT3 and MEK/ERK1/2 pathways. Further, treatment with Ruxolitinib (JAK2/STAT3 inhibitor) or Trametinib (MEK/ERK1/2 inhibitor) significantly enhanced the response of NB tumors to etoposide^[166]^. The ECM small leucine-rich proteoglycans (SLRPs), including decorin (DCN) and lumican (LUM), exhibited acquired upmodulation during NB-CSC enrichment^[146]^. Further, these small leucine rich proteoglycans (SLRP)-positive NB-CSCs were highly resistant to temozolomide (TMZ), demonstrating that (1) CSCs promote huge quantities of DCN and LUM; and (2) increased SLRPs promote acquired TMZ resistance, cellular heterogeneity, and a quiescent phenotype. The outcomes of this study clearly identified the pivotal role of SLRPs in drug resistance, the cell plasticity of NB-CSCs that dictates cell survival, and ECM/ TME modulation potential^[146]^.

### CSC-targeted therapy

**Figure 2:**
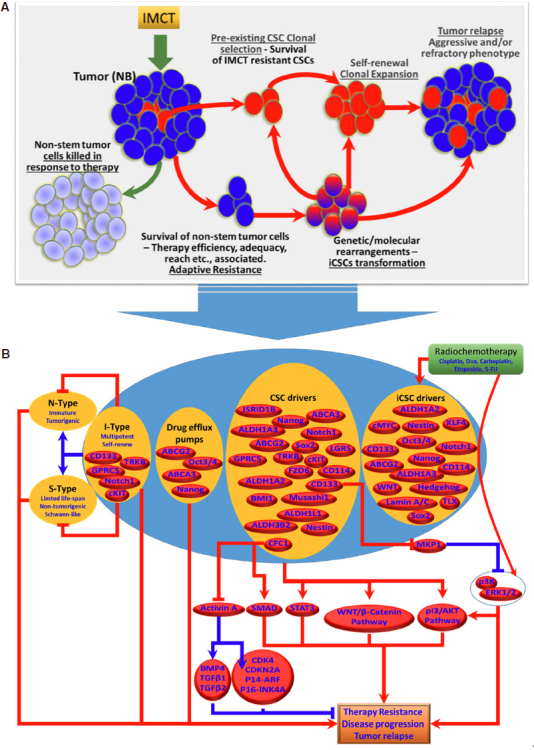
A: Cartoon showing the models of NB-CSCs-associated tumor resistance and tumor relapse. Pre-existing NB-CSCs survive IMCT and undergo self-renewal, clonal expansion and development of non-stem tumor cells, resulting in tumor relapse. In parallel, the non-stem tumor cells that survive IMCT under unique circumstances undergo extensive genetic and molecular rearrangements that lead to their transformation into induced CSCs (iCSCs). Generation of iCSCs with unique stem-cell characteristics of self-renewal and expansion results in tumor maintenance and relapse. B: Schematic representation of the molecular characteristics of NB-CSCs (pre-existing NB-CSCs and induced iCSCs) and their signaling flow-through that dictates therapy resistance and NB disease evolution. IMCT: intensive multi-modal clinical therapy; NB: neuroblastoma; CSCs: cancer stem cells; iCSCs: induced cancer stem cells; N-Type: neuroblastic type cells; I-Type: intermediate type neuroblastoma cells; S-Type: Schwann-type cells

Considering the significance of targeting CSCs or the formation of iCSCs in countering acquired therapy resistance and in the treatment of high-risk aggressive NB [Figure 2], recent studies are appropriately focused on developing improved therapeutic strategies. These proposed strategies include targeting specific surface markers, modulation of signaling pathways, adjustment of the microenvironment signals, inhibition of drug-efflux pumps, manipulation of miRNA expression, and induction of CSCs apoptosis and differentiation. Immunotherapy is often used in combination with chemotherapy and radiotherapy. It involves the use of antibodies that target specific cancer stem cell markers^[49]^. However, the limitations must be considered when selecting the appropriate target surface markers, as most of the documented markers are (1) not ubiquitously expressed in all (100%) NB-CSCs; and (2) many CSC markers are also expressed in normal stem cells^[60,74,167]^. Researchers have focused in identifying and characterizing agents [Rapamycin, dequalinium analogue, C-14 linker (DECA-14)] that could selectively target CSCs while sparing normal stem cells. Differentiating agents, such as RA alone or in combination with proteasome inhibitor (MG132), have been shown to decrease stem cell markers (Nestin, Sox2, Oct3/4) and suppress spheroid formation^[168,169]^. Recently, Bahmad *et al.*^[170]^ showed that targeting AKT/MTOR signaling in NB-CSCs could be beneficial. They reported that triciribine and rapamycin, which inhibit at 2 different points of the AKT/MTOR pathway, decrease cell survival and tumorosphere formation. Further, virotherapy can be an effective approach to kill CSCs. A study by Mahller *et al.*^[61]^ showed that engineered oncolytic virus Nestin-targeted oHSV significantly killed DOX-resistant CD133^**+**^ NB-SP. Interestingly in this study authors demonstrated the existence of CD34 and CD133 double positive cells within all patient derived cell-lines. The tumorospheres derived from these cells exhibited CD133 and ABCG2 enrichment, multi-lineage potential and are resistant to Doxorubicin. In an attempt to kill both the differentiated and tumor initiating NB cells, this study demonstrated that a nestin targeted engineered clinically safe oncolytic virus (rQNestin34.5) promoted a profound cell death of both bulk and tumor initiating tumorsphere-derived NB cells. More importantly, this study showed new virus production within in tumorsphere, and prompted a significant delay in tumor formation *in vivo*^[61]^*.* Induced telomerase activity (TA) also directly relates to clinical management of NB. Interestingly, studies found that the TA is selectively confined to the CSC-SP (CD15^**+**^) and is not detectable in most of the non-stem cancer cells and normal tissue stem cells^[171]^. Such a finding designates the TA as highly specific, and it could serve as a suitable candidate for anti-CSC therapy. It has also been shown that telomerase inhibition with Imetelstat will result in CSC exhaustion through its ability to irreversibly alter self-renewal capacity and cell growth^[171]^.

The FDA approved drug vorinostat (histone deacetylase inhibitor) increases chemosensitization, inhibits NB-CSCs’ tumorosphere formation capacity, reduced tumor cell invasion and deplete SP^[172]^. Further in doxorubicin-resistant NB-CSCs, vorinostat treatment resulted in the regulation of stemness maintenance and drug-resistance candidates including ABCB1, ABCC4, LMO2, SOX2, ERCC5, S100A10, IGFBP3, TCF3, and VIM^[172]^. Like-wise, the inhibition of L1-Cam (CD171), the driver for tumorigenic invasion and motility^[173]^ in CD133^**+**^ cells showed reduced cell survival, tumorosphere formation, self-renewal capacity, increased apoptosis and, suppressed tumor growth *in vivo*^[174]^*.* It was indicated that the effect is through Olig2 regulation and parallel activation of the tumor suppressor, p21 (WAF1/CIP1). In NB, studies have documented that CE7 epitope of L1-CAM is an useful tool in CAR-T targeted therapy^[175,176]^. The safety of targeting the CE7 epitope CD171 with CE7-CAR T cells has also been realized and, more importantly the potential to generate bioactive CAR-T cells from patients with recurrent/refractory disease after current IMCT^[176]^. Small molecule kinase inhibitor screening by Grinshtein *et al.*^[177]^ identified Polo-like kinase 1 (PLK-1) as the potential target for NB tumor initiating cells. To that end, a study by Pajtler *et al.*^[178]^ demonstrated that imidazotriazine (GSK461364), a competitive inhibitor for ATP binding to PLK1 which is in clinical development promoted cell death, inhibited clonal expansion and exerted anti-tumoral activity *in vivo*.

Along the line of targeting select CSC markers for NB cure, number of potential drug candidates are in the developmental stages and/or undergoing functional (molecular) characterization. For instance, Naveen *et al.*^[179]^ recognized the potential of Berberine, a plant alkaloid in EMT reversal, inhibiting cancer stemness, and prompting the neuronal differentiation. In NB, Berberine exerts programmed (DAXX, p53, *etc.*, dependent) cell death and inhibits tumor cell growth^[180-182]^. Berberine has been shown to revert EMT by inhibiting vimentin and fibronectin (mesenchymal markers) and restore E-cadherin, laminin and Smad^[179]^. Further this study identified the Berberine associated EMT reversal is through the downregulation of PI3/Akt and Ras-Raf-ERK signalling and subsequent upregulation of p38-MAPK. More importantly Berberine promotes neuronal differentiation as evidenced with upmodulation of MAP2, β-III tubulin and NCAM. In another study, Tian *et al.*^[183]^ recognized the clonal enrichment of NB-CSCs (CD133^**+**^) with chemodrug (etoposide) identified the potential of XAV939 a small molecule tankyrase (TNKS) inhibitor in alleviating the stemness physiognomy and migration of NB-CSCs. Cyclopamine (11-deoxojervine), a steroidal alkaloid (teratogen) derived from corn lily exerted a dramatic decrease in the CD133 and CD15 double positive populations that preserve more stemness characteristics^[184]^. Further, it has been shown that cyclopamine targets autocrine activation of hedgehog signaling and hence affect survival, clonal expansion and tumorosphere formation^[184]^. Like-wise, CD114^**+**^ NB-CSCs that exhibit high tumorigenicity, self-renewal capacity, and evasive phenotype has always remained as an attractive therapeutic candidate for stem-cell targeted therapy. In this regard, it has been shown that CD114 anti-body therapy and/or targeting its downstream driver STAT3 depletes CSC subpopulation within NB and corresponded with reduced metastatic state, increased chemosensitization and decreased tumor growth^[110]^. Targeting STAT3 has been considered for NB cure by many investigators and numerous promising candidates were investigated. For instance, a study from Goel and Aggarwal^[185]^ indicated that curcumin, a natural STAT3 inhibitor from turmeric promoted chemosensitization in multifarious tumors including NB. In addition, Cucurbitacin I, a triterpenoid that acts as a potent inhibitor of the STAT3/JAK has been shown to exert anti-tumor effect in NB^[186]^. Similarly, Honokiol, a biphenolic compound derived from the magnolia bark has been shown to target STAT3 signaling pathway and inhibit NB growth^[187]^. Owing to the criticality of developing effective drug deliverables in targeting tumor initiating CSCs, their clonal enrichment, resistance to current IMCT, the acquired formation of iCSCs with IMCT, investigations around the globe are appropriately focused in identifying/characterizing promising drug candidates. See Table 2 for the summation of a list of such candidates in the pipeline and are characterized. Identifying novel adjuvants that selectively deplete CSCs or target the formation of iCSCs could lead to the development of improved therapeutic strategies for the cure NB.

**Table 2:** Short list of drug candidates that were investigated for their efficacy in killing and/or differentiation of NB-CSCs

Drug	Targets	Ref.
Retinoic Acid + proteasome inhibitor MG132	Nestin, Sox2, Oct4	Hämmerle *et al.*^[168]^
Dequalinium analogue, C-14	Metabolic Pathways	Smith *et al.*^[169]^
Rapamycin	p70S6K, S6RP	Smith *et al.*^[169]^
Rapamycin	AKT	Bahmad *et al.*^[170]^
Triciribine	mTOR	Bahmad *et al.*^[170]^
Oncolytic virus	Nestin	Mahller *et al.*^[61]^
Imetelstat, RNA TR oligonucleotide antagonist	hTERT	Castelo-Branco *et al.*^[171]^
Vorinostat, histone deacetylase inhibitor	ABCB1, ABCC4, LMO2, SOX2, ERCC5, S100A10, IGFBP3, TCF3, VIM	Zheng *et al.*^[172]^
imidazotriazine (GSK461364)	PLK-1	Pajtler *et al.*^[178]^
Berberine	Vimentin, fibronectin, E-cadherin, laminin, Smad, PI3/Akt, Ras-Raf-ERK	Naveen *et al.*^[179]^
XAV939 a small molecule tankyrase (TNKS) inhibitor	TNKS, CD133	Tian *et al.*^[183]^
Cyclopamine (11-deoxojervine)	Hedgehog signaling	Schiapparell *et al.*^[184]^
Curcumin	Stat3	Goel and Aggarwal^[185]^
Cucurbitacin I (triterpenoid)	STAT3/JAK	Gheeya *et al.*^[186]^
Honokiol (biphenolic compound)	STAT3 signaling	Prasad *et al.*^[187]^
Aqueous ethanolic extract of T. cordifolia	NFkB, NCAM, MMPs	Mishra and Kaur^[189]^
Aspirin (acetylsalicylic acid)	p21Waf1, hypo-pRb1	Pozzoli *et al.*^[190]^
Rexinoid + IIF + EGCG	MMP-2, MMP-9 and COX-2	Farabegoli *et al.*^[191]^

Drugs (combinations) tested, targeted molecular drivers and the studies are listed. A complete list if CSC-targeting compounds, mode of action in and beyond NB is discussed in detail elsewhere^[188]^

On the current clinical perspective for NB CSC-targeted therapy, autologous stem-cell rescue after myeloablative chemotherapy with/without radiotherapy has been shown to relatively improve patient survival^[192,193]^. Further, studies have indicated the effectiveness of high-dose therapy with tandem or triple autologous stem-cell rescue in treating high-risk NB, with encouraging long-term survival, slashed CNS relapse and secondary malignancies^[194,195]^. The benefit of such a major therapeutic advancement is limited by the risk of reinfusing NB cancer cells that could lead to and/or attributable to the post-transplant relapse^[196,197]^. However, significant number of strategies are currently under consideration or revisited to betterment the purging efficacy that could benefit in long-term clinical outcome for NB. The strategy, effect, clinical practice, modifications and pitfalls are adequately documented/reviewed elsewhere^[198-202]^ and hence is not discussed in this review.

## Conclusions and perspectives

The existence, clonal selection, and enrichment of CSCs contributes to NB disease progression, resistance to therapeutic measures, and poor prognosis [Figure 2A]. It has been widely recognized that the treatment modalities that spare these CSC clones allow them to self-renew and recapitulate the non-stem tumor cell mass, subsequently leading to tumor relapse. In addition, the outcomes of recent investigations have recognized the formation of iCSCs and their influence in treatment resistance and disease evolution. It is apparent that some, if not all, current treatment components (radiation, chemotherapy drugs) inflict acquired genetic and molecular rearrangements in non-stem tumor cells and prompt the transformation into iCSCs that are extremely tumorigenic and equipped with self-renewing capacity, stemness maintenance, and drug resistance [Figure 2A]. It is critical to develop therapeutic measures that target both the pre-existing CSCs and the iCSCs, if we are to counter therapy resistance and successfully treat NB. Although a number of promising CSC surface markers for NB have been characterized and their cellular and molecular functions in stemness, therapy response, and disease progression have been realized (schematically summarized in Figure 2B), two crucial factors, (1) the lack of ubiquitous availability of specific candidate in all NB-CSCs within the tumor and (2) the presence of candidate marker(s) in normal non-tumorigenic stem cells highly limit their use in developing a CSC-targeted approach. Preliminary studies of the CSC-targeted approach to counter therapy resistance and as an adjuvant with current clinical therapy are encouraging. We believe that this review will provide an up-to-date understanding of NB-CSCs in disease evolution and drug resistance*.* Overall, the documented evidence supports the enormous clinical potential of targeting CSCs to counter therapy resistance and disease evolution, and warrants further rigorous investigation.

## Declarations

### Copyright

© The Author(s) 2019.
